# Carbon Nanomaterials Based Electrochemical Sensors/Biosensors for the Sensitive Detection of Pharmaceutical and Biological Compounds

**DOI:** 10.3390/s150922490

**Published:** 2015-09-04

**Authors:** Bal-Ram Adhikari, Maduraiveeran Govindhan, Aicheng Chen

**Affiliations:** Department of Chemistry, Lakehead University, 955 Oliver Road, Thunder Bay, ON P7B 5E1, Canada; E-Mails: badhikar@lakeheadu.ca (B.-R.A.); mgovindh@lakeheadu.ca (M.G.)

**Keywords:** SWCNTs, graphene, buckypaper, methylglyoxal, NADH, glucose, acetaminophen, valacyclovir

## Abstract

Electrochemical sensors and biosensors have attracted considerable attention for the sensitive detection of a variety of biological and pharmaceutical compounds. Since the discovery of carbon-based nanomaterials, including carbon nanotubes, C_60_ and graphene, they have garnered tremendous interest for their potential in the design of high-performance electrochemical sensor platforms due to their exceptional thermal, mechanical, electronic, and catalytic properties. Carbon nanomaterial-based electrochemical sensors have been employed for the detection of various analytes with rapid electron transfer kinetics. This feature article focuses on the recent design and use of carbon nanomaterials, primarily single-walled carbon nanotubes (SWCNTs), reduced graphene oxide (rGO), SWCNTs-rGO, Au nanoparticle-rGO nanocomposites, and buckypaper as sensing materials for the electrochemical detection of some representative biological and pharmaceutical compounds such as methylglyoxal, acetaminophen, valacyclovir, β-nicotinamide adenine dinucleotide hydrate (NADH), and glucose. Furthermore, the electrochemical performance of SWCNTs, rGO, and SWCNT-rGO for the detection of acetaminophen and valacyclovir was comparatively studied, revealing that SWCNT-rGO nanocomposites possess excellent electrocatalytic activity in comparison to individual SWCNT and rGO platforms. The sensitive, reliable and rapid analysis of critical disease biomarkers and globally emerging pharmaceutical compounds at carbon nanomaterials based electrochemical sensor platforms may enable an extensive range of applications in preemptive medical diagnostics.

## 1. Introduction

Owing to the advantages of instrumental simplicity, moderate cost, and portability, electroanalytical techniques provide a powerful sensing strategy, superior to various traditional analytical methods [1]. Assorted electroanalytical techniques have been employed for the development of electrochemical sensors and biosensors for the detection and quantification of myriad biomarker species, chemical compounds, and minerals in environmental and biological samples [2,3,4]. The development of active electrocatalysts plays a key role in the design of efficient, reliable, stable, and innovative sensing devices [5]. Numerous electrochemical sensor platforms have been developed for the detection and quantification of medically and pharmaceutically important compounds such as glucose, methylglyoxal, nicotinamide adenine dinucleotide (NADH), acetaminophen, and valacyclovir. Moreover, recent developments in the field of nanotechnology and materials science have paved the way for the synthesis of many new materials with desired morphologies and unique physicochemical properties [6].

Carbon-based nanomaterials such as carbon nanotubes, graphene, buckypaper, and nanohybrids among various other types of engineered nanomaterials have received enormous attention due to their promising sensor applications, which are most widely used in the electrochemical sensing of various compounds [7,8,9]. Carbon nanomaterials offer diverse advantages with their unique properties, such as a high surface-to-volume ratio, high electrical conductivity, chemical stability, biocompatibility, and robust mechanical strength [10,11,12]. Carbon possesses the capacity to hybridize into sp, sp^2^ and sp^3^ configurations with narrow gaps between their 2s and 2p electron shells. These unique properties are responsible for enabling the design of versatile carbon-based nanomaterials for the sensitive detection of biological compounds [13,14,15]. Carbon nanotubes may be comprised of a single graphitic layer, or multiple coaxial layers, resulting in the formation of single-walled carbon nanotubes (SWCNTs) and multiple-walled carbon nanotubes (MWCNTs). SWNTs are seamlessly wrapped into cylindrical tubes, having diameters of between 0.4 nm and 2.5 nm. These materials offer excellent physical and chemical properties that enable a wide range of applications in biomedicine [16].

Methylglyoxal is a predictor in type 2 diabetes mellitus of intima-media thickening, vascular stiffening, and the elevation of systolic blood pressure, suggesting its clinical usefulness as a biomarker for diabetic macroangiopathy [17]. In view of the prominence of methylglyoxal in clinical applications, there are many methods reported for its determination. The use of SWCNTs as a sensing material dramatically decreases the overpotential, which makes a promising carbon nanomaterial for the detection of variety of biological compounds. Chen and co-workers have demonstrated a SWCNT based sensor platform for the quantitative determination of methylglyoxal in 0.1 M PBS (pH 7.4), showing that the sensor system was highly specific and sensitive for the detection of plasma levels of methylglyoxal in healthy individuals and diabetic patients [18].

Valacyclovir is the drug of choice for the treatment of herpes zoster and cold sores, and is an l-valyl ester and prodrug of the antiviral drug acyclovir. Subsequent to absorption, valacyclovir is rapidly and almost completely hydrolyzed to acyclovir and l-valine, which is an essential amino acid. It is also effective for the suppression or treatment of genital herpes in immunocompetent individuals. SWCNTs have been utilized to modify GCE for the sensitive detection of valacyclovir [19].

Graphene is a much newer member in the family of carbon materials in comparison to fullerenes. Graphene is an atomically thin film that consists of hexagonally arranged carbon atoms with sp^2^ hybridization in two dimensions [15]. The combination of attributes such as a high surface area, enhanced mobility of charge carriers, and high stability makes graphene an ideal platform for the anchoring of metal nanoparticles for electrochemical sensing applications [20,21,22]. NADH/NAD^+^ is an important co-enzyme couple that plays a significant role in energy production/consumption within the cells of living organisms, which participates in a variety of enzymatic reactions involving more than 300 dehydrogenases [23]. Acetaminophen is a widely used analgesic (pain reliever) and fever reducer, and is considered to be very safe when administered at recommended dosages; however, it causes hepatotoxicity at higher doses [24]. Recently, graphene and Au nanoparticle-rGO nanocomposite based electrochemical sensors have been fabricated by Chen and co-workers [25,26] via an electrochemical method for the sensitive detection of NADH and acetaminophen.

Buckypaper is a thin film (5–25 µm) comprised of lateral arranged networks of nanotubes, which constitute a promising platform for the fabrication of highly concentrated and aligned nanotube-reinforced composites. Recently, much attention has been given to macroscopic assemblies of carbon nanotubes, which include carbon buckypapers, in order to exploit the characteristic properties of single carbon nanotubes at macroscopic scales [27]. Buckypapers and composites thereof are innovative materials with intriguing physical and chemical properties that have strong potential for pharmacological and prosthetic applications. Buckypaper has been incorporated into the design of a mediator-free glucose sensor with high sensitivity, stability, selectivity, and reproducibility [28].

This feature article highlights some recent development of electrochemical sensor platforms employing SWCNTs, rGO, SWCNT-rGO nanocomposites, Au nanoparticle-rGO nanocomposites, and buckypapers as sensing materials for the sensitive electrochemical detection of methylglyoxal, acetaminophen, valacyclovir, NADH and glucose with a main focus of the work carried out in the authors’ group. The conceptualization, design, optimization, and electrocatalytic activity of carbon based nanomaterials and nanocomposites for the detection of various important biological and pharmaceutical compounds are also described to demonstrate the promising medical applications of the carbon nanomaterial based electrochemical platforms.

## 2. Experimental

### 2.1. Chemicals and Reagents

All chemicals used in experiments, including methylglyoxal, acetaminophen, valacyclovir, glucose oxidase (GOx) (EC 1.1.3.4), horseradish peroxidase (HRP), β-D-glucose, NADH, graphene oxide (4 mg/mL), SWCNTs and chitosan, were purchased from Sigma-Aldrich (St. Louis, MO, USA). The buckypaper employed in this study was produced by the High-Performance Materials Institute at Florida State University (Tallahassee, FL, USA), with a thickness of 0.035 mm. All experiments were performed in a 0.1 M phosphate buffer solution (PBS) prepared with Na_2_HPO_4_ and NaH_2_PO_4_. Titanium foils (99.2%) were purchased from Alfa Aesar (Ward Hill, MA, USA). All chemicals were used as received without further purification. All solutions were prepared with pure water produced by a Nanopure^®^ water purification system. All solutions were freshly prepared and used within 24 h.

### 2.2. Fabrication of Electrochemical Sensors

Prior to modification, a glassy carbon electrode (GCE) was polished with 0.05 μm alumina powders, then sonicated for 3 min in the pure water, and finally allowed to dry at room temperature [26]. Similarly, titanium plates (1.25 cm × 0.8 cm × 0.5 mm) were ultrasonically cleaned in acetone, ethanol and water [28]. For the fabrication of the SWCNT/GCE, a 0.5 mg/mL suspension was prepared by dispersing 0.5 mg SWNTs in 1.0 mL N,N-dimethylformamide via ultrasonic agitation. The GCE surface was then coated with a known volume (40 μL) of this suspension and allowed to dry at room temperature [18,19]. For the rGO/GCE, a 4 mg/mL GO solution was added to a 0.1 M (pH 9) phosphate buffer solution (PBS) to dilute a 0.3 mg/mL GO colloidal dispersion followed by 30 min of sonication. The GO suspension within the electrochemical cell was deoxygenated using Ar gas for 15 min. Cyclic voltammetric deposition was performed in the GO suspension (0.3 mg/mL) with an electrode potential range between 0.5 to −1.5 V at scan rate of 10 mV/s using a three-electrode system with a bare GCE as the working electrode. Five reduction cycles were employed in this study to deposit rGO onto the GCE surface [26]. The Au nanoparticle-rGO/GCE was fabricated by casting a 5 µL mixture of GO (0.5 mg/mL), AuCl_3_ (10 mM), and Nafion (0.5%) on the cleaned GC electrode, which was then allowed to air dry.

**Scheme 1:**
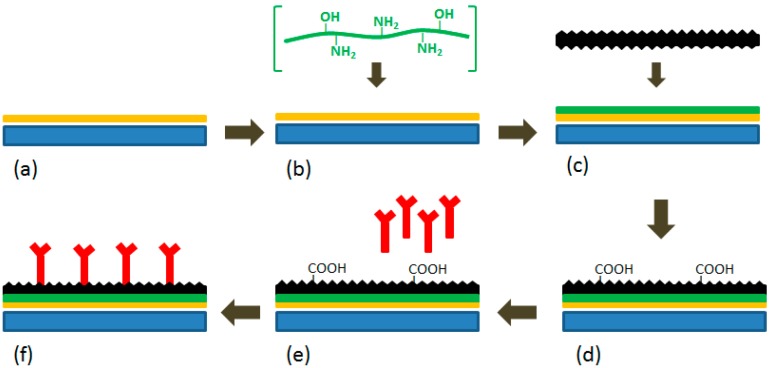
Schematic diagram of the preparation of the buckypaper-based electrochemical biosensor. (**a**) a titanium plate sputtered with a thin layer of gold; (**b**) gold interaction with chitosan; (**c**) attachment of buckypaper to the gold surface via chitosan; (**d**) activation of the buckypaper; (**e**) immobilization of enzymes; (**f**) the fabricated biosensor. All figures are reproduced with permission from [28]. Copyright 2011 Elsevier.

The *in situ* formation of Au nanoparticle-rGO sheets on the GCE was achieved in 0.1 M H_2_SO_4_ by applying a potential of −1.0 V (*vs.* Ag/AgCl) for 500 s [25]. The SWCNT-rGO nanohybrid/GCE was fabricated by casting 40 μL (0.5 mg/mL) of each batch of SWCNTs prepared in N,N-dimethylformamide and GO suspension (4 mg/mL) on the GCE surface, followed by air drying and electrochemical reduction of GO through continuous cycles under a potential range between 0.5 and −1.5 V for five cycles. Details on the fabrication of the buckypaper based biosensor are presented in Scheme 1 [28]. The titanium electrode for glucose sensing was prepared by attaching a portion of the buckypaper to a gold coated titanium plate, which was facilitated by 5 µL of 2 mg/mL chitosan. The buckypaper surface was then activated through cyclic voltammetry with a potential range between −0.8 V and 0.4 V at a scan rate of 10 mV/s in a phosphate buffer solution (PBS) followed by 90 s amperometric scanning at a potential of 1.5 V in order to generate carboxylic groups. The activated surface was then enzymatically immobilized via the casting of a mixed solution comprised of 20 µL of 5 mg/mL GOx, 10 µL of 2 mg/mL HRP and 10 µL of 2 mg/mL chitosan onto the buckypaper.

### 2.3. Apparatus

All electrochemical experiments, including cyclic voltammetry (CV), differential pulse voltammetry (DPV), square wave voltammetry (SWV) and amperometry were conducted with a CHI 660D electrochemical workstation (CH Instruments Inc., Austin, TX, USA) using a conventional three-electrode system that consisted of a platinum wire counter electrode, a 3 M KCl saturated Ag/AgCl reference electrode, and a carbon nanomaterial based working electrode. A field-emission scanning electron microscope (FE-SEM; Hitachi SU-70, Japan) was employed to characterize the surface morphologies of the carbon nanomaterials and their nanocomposites, which were deposited on the surfaces of the electrode. All experiments were performed at room temperature and the electrode potentials quoted are *versus* an Ag/AgCl electrode.

## 3. Results and Discussion

### 3.1. Electrochemical Sensing of Methylglyoxal, Acetaminophen and Valacyclovir at SWCNTs

**Figure 1:**
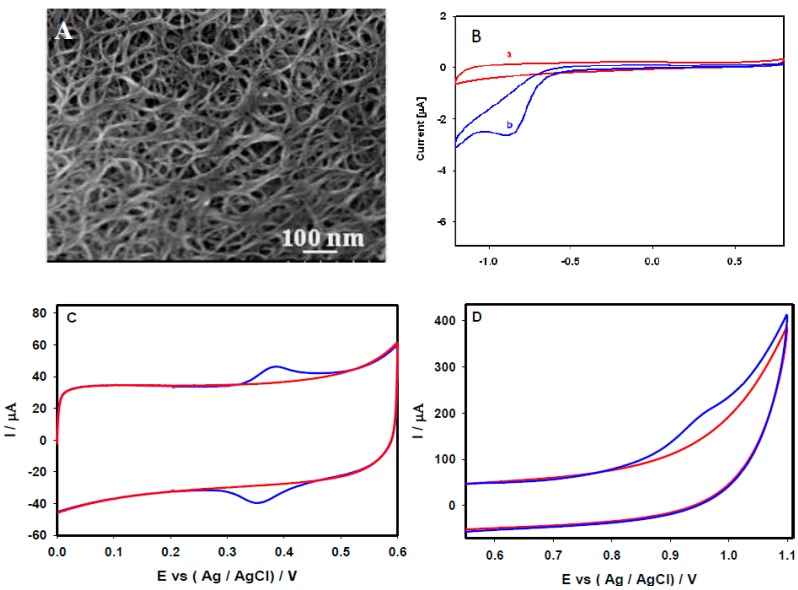
(**A**) FE-SEM images of the SWCNTs on the GCE surface. CV performance recorded at the SWCNT/GCE, in the absence (red solid line), and in the presence of (blue solid line) (**B**) 10 µM methylglyoxal, (**C**) 50 µM acetaminophen, and (**D**) 50 µM valacyclovir, in 0.1 M PBS (pH 7.4) at a scan rate of 20 mV/s. Figure 1B reproduced with permission from [18]. Copyright 2013 Elsevier.

SEM was utilized to study the surface characteristics and morphology of the fabricated SWCNT-modified GCE. Figure 1A depicts a SEM image of the SWCNTs on the GCE surface, which were uniformly covered with an interlinked nanoporous network structure. The prepared SWCNT/GCE was tested for the detection of methylglyoxal, acetaminophen, and valacyclovir. Figure 1B shows the cyclic voltammograms (CVs) of the SWCNT/GCE recorded in 0.1 M PBS (at pH 7.4) at a scan rate of 20 mV/s in the absence (Curve a) and presence of 10 μM methylglyoxal (Curve b). A single well-defined reduction peak of methylglyoxal was observed at −0.864 V (Curve b) compared with the CV recorded in 0.1 M PBS (Curve a). The absence of anodic peaks in the reverse scan revealed that the reduction of methylglyoxal in this particular system was irreversible in nature.

Figure 1C depicts a typical reversible behavior of 50 μM acetaminophen at the SWCNT/GCE 0.1 M PBS at pH 7.4 under a scan rate of 20 mV/s. A distinct oxidation peak at 0.372 V and reduction peak at 0.355 V obtained during the measurement of CV revealed its reversible behavior. As shown in Figure 1D, the CV of 50 μM valacyclovir in 0.1 M PBS (pH 7.4) at the scan rate of 20 mV/s generated distinct broad oxidation peak at 0.978 V, whereas no obvious reduction peak was observed during reverse scanning, indicating the irreversible catalytic behavior of valacyclovir.

**Figure 2:**
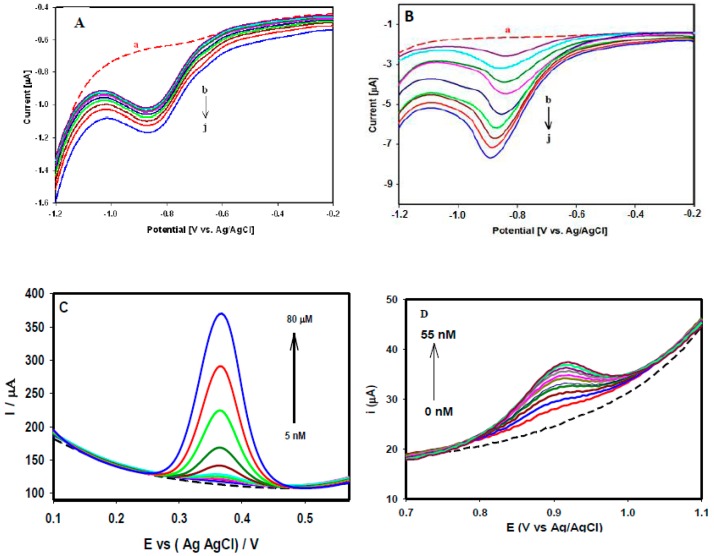
Responses of the SWCNTs/GCE recorded in 0.1 M PBS (pH 7.4) (**A**) and (**B**) SWV responses of different methylglyoxal concentrations (0.1 µM to 100 µM) (**C**) DPV responses of acetaminophen (5 nM to 80 µM) and (**D**) DPV responses of valacyclovir (5–55 nM). Figure 2A,B reproduced with permission from [18]. Copyright 2013 Elsevier. Figure 2D reproduced with permission from [19]. Copyright 2013 Royal Society of Chemistry.

Figure 2A,B illustrate the responses of various concentrations of methylglyoxal through square wave voltammetry (SWV), where the peak current was increased linearly with the successive addition of different concentrations of methylglyoxal. Figure 2A depicts a linear increase in the peak current responses with increased methylglyoxal concentrations in the range 0.1–2.0 μM, with a strong linear association (R^2^ value of 0.99). Similarly, Figure 2B shows the responses of the higher concentration range of (20–100 μM). Different concentrations of acetaminophen were studied through DPV as presented in Figure 2C, where the peak current increased linearly with increasing concentrations from 5 nM to 80 μM with a linear association (R^2^) value of 0.97 and a calculated limit of detection of 4.3 nM. Figure 2D shows the DPV responses of different valacylovir concentrations varied from 5 nM to 55 nM with a limit of detection of 1.8 nM.

Besides the aforementioned three examples, carbon nanotubes and carbon nanotube composite based electrochemical sensing platforms have been widely explored for the detection of various biological and pharmaceutical compounds including glucose [29,30,31], lactate [32], dopamine [33,34,35], rutine [36], human serum albumin [37], DNAs [38], epinephrine [39,40], cholesterol [41,42], methimazole [43] , sumatriptan [44], and paracetamol [45]. The electrochemical methods, the linear range and the detection limit of those studies are compared in Table 1.

**Table 1:** List of electrochemical sensors and biosensors based on carbon nanomaterials and their composites for the detection of biological and pharmaceutical compounds.

Carbon Based Nanomaterials	Analytes	Methods	Linear Range	Detection Limit	Reference
SWNT-Nafion-GOx	Glucose	Amperometry	2 mM to 20 mM	-	29
SWNT-GOx	Glucose	Amperometry	Up to 40 mM	-	30
Pt-Nafion-SWCNTs-GOx	Glucose	Amperometry	0.5 µM to 5 mM	0.5 µM	31
SWNT-mineral-oil paste	Lactate	Amperometry	Up to 7.0 mM	0.3 mM	32
Nafion-SWNT	Dopamine	DPV	0.02 µM to 6.0 µM	5.00 nM	33
SWNT polymer composite	Dopamine	CV	16 nM to 600 µM	8 nM	34
SWCNTs	Dopamine	DPV	3 µM to 200 µM	48 nM	35
SWCNTs	Rutine	CV	20 nM to 5 µM	10 nM	36
SWCNTs	Human serum albumin	CV	0.075 nM to 7.5 nM	75 pM	37
SWCNTs	DNAs	DPV	5 µM to 30 µM	1.43 µM	38
MWCNTs-Nafion	Epinephrine	CV and DPV	0.06 mM to 0.24 mM	0.02 mM	39
MWNT nanocomposite	Epinephrine	LSV	50 nM to 10 µM	10 nM	40
MWCNTs	Cholesterol	Amperometry	Up to 6.0 mM	0.2 mM	41
MWCNTs	Cholesterol	Amperometry	100 mg/dL to 400 mg/dL	-	42
MWCNTs	Methimazole	Amperometry	0.074 µM to 63.5 µM	0.056 µM	43
MWCNTs-silver nanoparticles	Sumatriptan	CV	80 nM to 100 µM	40 nM	44
MWCNTs	Paracetamol	ASV	0.01 µM to 20 µM	10 nM	45
SWCNTs	Methylglyoxal	SWV	0.1 µM to 100 µM	-	18
SWCNTs	Valacyclovir	DPV	5 nM to 55 nM	1.8 nM	19
SWCNTs	Acetaminophen	DPV	5 nM to 80 µM	4.3 nM	This work
CuO-graphene	Glucose	Amperometry	1 µM to 8 mM	1 µM	46
CuNPs/graphene	Glucose	Amperometry	0.5 µM to 4.5 mM	0.5 µM	47
Graphene-ppy	Glucose	Amperometry	-	3 µM	48
Graphene-Pt	Ascorbic acid	DPV	0.03 µM to 8.13 µM	0.03 µM	49
Graphene	Norepinephrine	Amperometry	0.04 µM to 100 µM	0.84 nM	50
Reduced GO	NADH	Amperometry	10 µM to 600 µM	0.33 µM	51
Graphene-Au nanorod	NADH	Amperometry	5 µM to 337 µM	1.5 µM	52
Au-TiO_2_/graphene	NADH	Amperometry	10 µM to 240 µM	0.2 µM	53
Graphene-TiO_2_	NADH	Amperometry	10 nM to 2 mM	3 × 10^−9^ M	54
AuNPs-rGO	NADH	Amperometry	50 nM to 500 µM	1.13 nM	25
Nitrogen doped Graphene	Uric acid	DPV	0.1 µM to 20 µM	0.045 µM	55
Graphene	Uric acid	Amperometry	0.19 µM to 49.68 µM	0.132 µM	56
Nafion-AgNPs-rGO	Uric acid	LSV	10 µM to 800 µM	8.2 µM	57
Pt-rGO	Uric acid	DPV	10 µM to 130 µM	0.45 µM	59
ERGO	Serotonin	DPV	5 µM to 300 µM	0.11 µM	59
ERG/Ni_2_O_3_-NiO	acetaminophen	DPV	0.04 µM to 100 µM	0.02 µM	60
Graphene-chitosan	Acetaminophen	DPV	1 µM to 100 µM	0.3 µM	61
Graphene	Acetaminophen	SWV	0.1 µM to 20 µM	0.032 µM	62
rGO	Acetaminophen	DPV	5 nM to 800 µM	2.13 nM	26
SWCNTs-GNS	Acetaminophen	DPV	0.05 µM to 64.5 µM	0.038 µM	63
MWCNT-graphene nanosheets	Acetaminophen	DPV	0.8 µM to 110 µM	0.1 µM	64
MWCNT/GO	Acetaminophen	DPV	0.5 µM to 400 µM	47 nM	65
SWCNTs-rGO	Acetaminophen	DPV	5 nM to 80 µM	1.4 nM	This work
MWCNT/GO	Dopamine	DPV	0.2 µM to 400 µM	22 nM	65
MWCNT/GONR	Dopamine	DPV	0.15 µM to 12.15 µM	0.08 µM	66
Buckypaper-SWCNTs	Glucose	Amperometry	0 mM to 10 mM	0.022 mM	70
Buckypaper-GOx-HRP	Glucose	Amperometry	Up to 9 mM	0.01 mM	28

### 3.2. Electrochemical Sensing of Acetaminophen and NADH at rGO and Au Nanoparticle-rGO Nanocomposites

**Figure 3:**
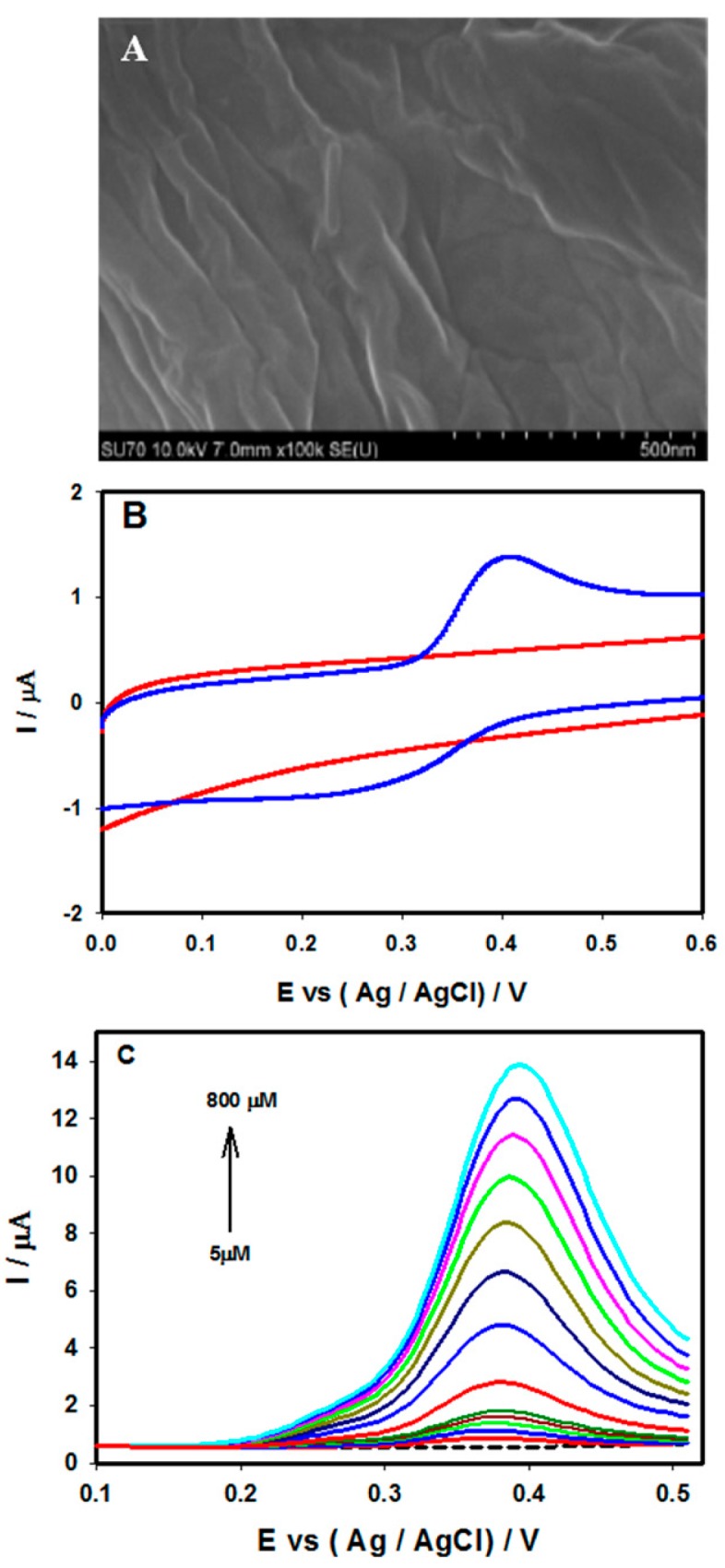
(**A**) FE-SEM images of electrochemically reduced grapheme oxide (rGO) on the GCE surface; (**B**) CVs recorded at the rGO/GCE in the absence (red solid line), and presence of 50 µM acetaminophen (blue solid line) in 0.1 M PBS (pH 7.4) at a scan rate of 20 mV/s; (**C**) DPVs of the rGO/GCE recorded in 0.1 M PBS (pH 7.4) containing different acetaminophen concentrations (5 µM to 800 µM). Figure 3A and 3B are reproduced with permission from [26]. Copyright 2015 Elsevier.

**Figure 4:**
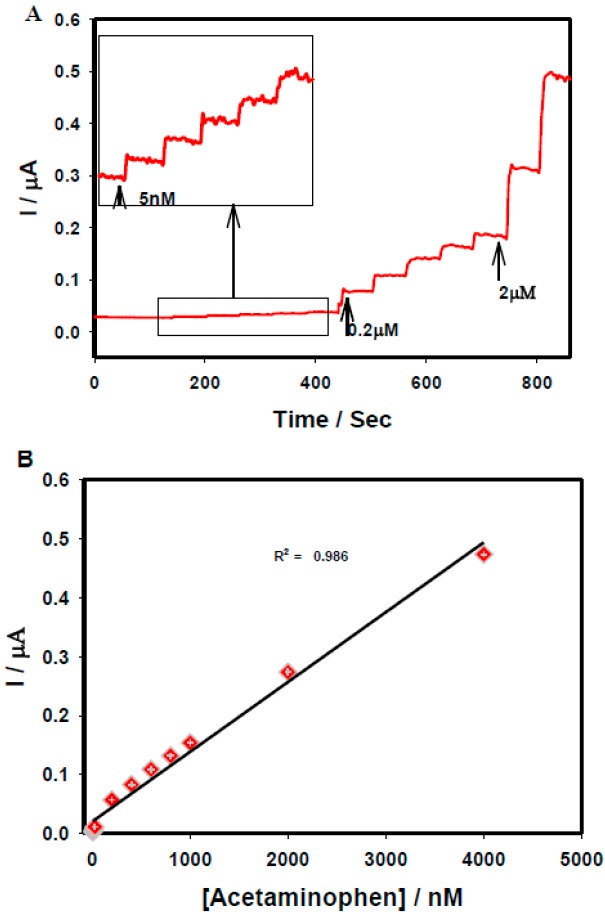
(**A**) Amperometric responses recorded at rGO/GCE in 0.1 M PBS under various acetaminophen concentrations (5 nM–4 µM), E_app_: 0.5 V and (**B**) the calibration plot of current vs acetaminophen concentrations. All figures are reproduced with permission from [26]. Copyright 2015 Elsevier.

Figure 3A displays a FE-SEM image of rGO sheets deposited on GCE through an electrochemical technique, where a characteristic rippled-like structure indicated that the GCE surface was uniformly coated with graphene. The electrochemical behavior of 50 µM acetaminophen was studied through CV at rGO/GCE in 0.1 M PBS at pH 7.4 at the scan rate of 20 mV/s (Figure 3B). The appearance of the redox peaks indicated a reversible electrode process. The performance of rGO/GCE toward different concentrations of acetaminophen was studied through differential pulse voltammetry (DPV). Figure 3C shows the linear increments of current responses upon the successive addition of acetaminophen concentrations from 5 µM to 800 µM with an excellent correlation coefficient (R^2^ = 0.996). The analytical performance of the rGO/GCE for the lowest acetaminophen concentrations were evaluated through an amperometric technique under a constant electrode potential of 0.5 V. A rapid increment of current was observed upon the successive addition of 5.0 nM, 0.2 µM, and 2.0 µM acetaminophen solutions in 20 mL of 0.1 M PBS (Figure 4A). The calibration plot of current vs acetaminophen concentration gave a strong linear association with a correlation coefficient of R^2^ = 0.986 (Figure 4B). The limit of detection (LOD) was calculated to be 2.1 nM using 3s/b, where s is the standard deviation of the blank and b is the slope of the calibration curve.

**Figure 5:**
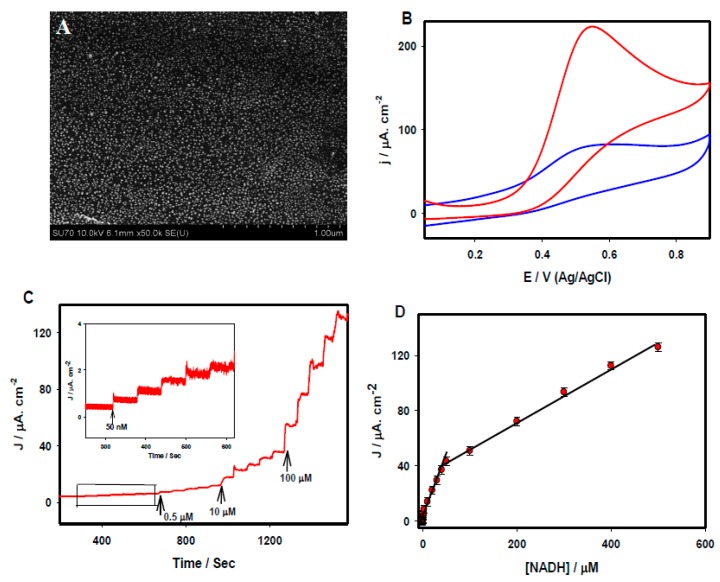
(**A**) FE-SEM images of the AuNP-rGO on the GCE surface. (**B**) CVs recorded at the rGO/GCE (blue), and AuNPs-rGO/GCE (red) recorded in 1 mM NADH. (**C**) Amperometric i-t curve recorded at the Au-rGO/GCE in 0.1 M PBS under various NADH concentrations (50 nM–0.5 mM), Eapp: 0.55 V and (**D**) the calibration plot of current density *vs*. NADH concentration. All figures are reproduced with permission from [25]. Copyright 2015 Elsevier.

Figure 5A shows a typical FE-SEM image of the Au nanoparticle/rGO nanocomposite electrode. Au nanoparticles with an average size of 8.2 nm were uniformly distributed across the rGO sheet. Figure 5B compares the CVs of the rGO/GCE (blue) and Au nanoparticle-rGO/GCE (red) recorded in 0.1 M PBS (pH 7.2) containing 1 mM NADH at the scan rate of 20 mV/s. A broad anodic peak appeared at 0.54 V for NADH at the rGO/GCE whereas a large well-defined anodic oxidation peak appeared at the Au nanoparticle-rGO/GCE, with a ~2.3 fold increase of the current density. Figure 5C displays amperometric i-t curves under an applied potential of 0.55 V for the sensitive detection of NADH using an Au nanoparticle-rGO/GCE platform. The calibration plot of current *vs.* NADH concentrations are presented in Figure 5D, which shows two linear concentration ranges: one from 50 nM to 50 µM with a correlation coefficient of R^2^ = 0.987, and the other from 50 µM to 500 µM with a correlation coefficient of R^2^ = 0.997. The limit of detection was calculated to be 1.13 nM. Such a low detection limit was far improved from those of most existing reports with graphene and their composite materials for the electrochemical detection of NADH. Moreover, a variety of electrochemical sensors were prepared with graphene and its nanocomposites for the detection of glucose [46,47,48], ascorbic acid [49], norepinephrine [50], NADH [51,52,53,54], uric acid [55,56,57,58], serotonin [59], and acetaminophen [60,61,62]. The performance of these electrochemical sensors is also compared in Table 1.

### 3.3. Electrochemical Sensing of Acetaminophen and Valacyclovir at SWCNTs-rGO Nanocomposites

**Figure 6:**
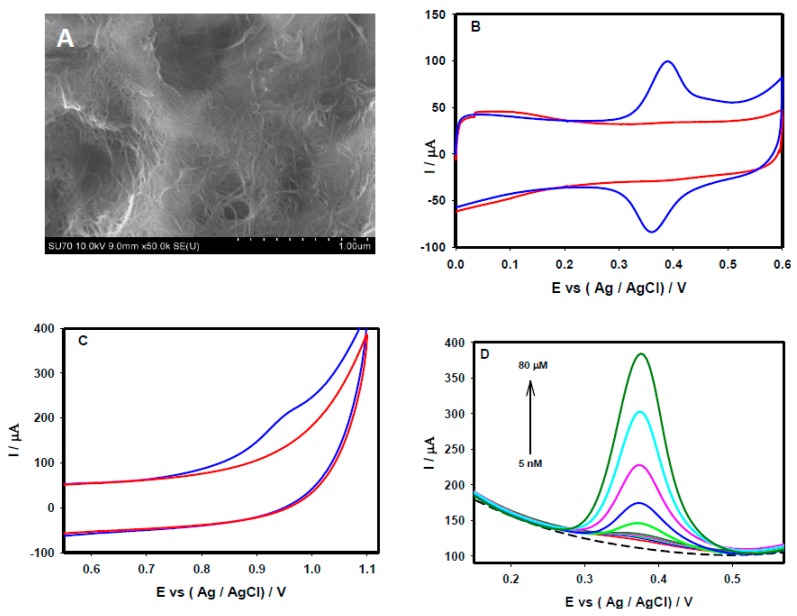
(**A**) FE-SEM images of the SWCNTs-rGO nanocomposite on the GCE surface. CV performance recorded at the SWCNT-rGO nanocomposite/GCE in the absence (red solid line), and presence of (blue solid line) (**B**) 50 µM acetaminophen, (**C**) 50 µM valacyclovir in 0.1 M PBS (pH 7.4) at a scan rate of 20 mV/s. (**D**) DPVs of the SWCNT-rGO nanocompoposite/GCE recorded in 0.1 M PBS (pH 7.4) containing different acetaminophen concentrations (5 nM to 80 µM).

Figure 6A depicts a FE-SEM image of the SWCNT-rGO nanocomposite, in which graphene films were uniformly dispersed throughout the SWCNTs. The electrochemical behaviors of the SWCNT-rGO/GCE were investigated through the CV response of 50 µM acetaminophen in 0.1 M PBS (pH 7.4) at the scan rate of 20 mV/s. As shown in Figure 6B, a distinct well-defined reversible peak (blue solid line) was observed, which can be attributed to the oxidation and reduction of acetaminophen. The current response observed through the oxidation and reduction of 50 µM acetaminophen at the SWCNT-rGO/GCE was much higher than that of the responses obtained through individual SWCNT (Figure 1C) and rGO (Figure 3B) platforms. Figure 6C shows the typical valacyclovir response at the SWCNTs-rGO/GCE with a distinct oxidation peak (blue solid line), having no obvious reduction peak during reverse scanning. DPV responses of different concentrations of acetaminophen at the SWCNT-rGO/GCE are presented in Figure 6D. The current responses were increased with increasing concentrations of acetaminophen (from 5 nM to 80 µM), and shows a linear association (R^2^ value of 0.97) between current *vs.* acetaminophen concentrations. The low detection limit of 1.4 nM was achieved at the SWCNT-rGO nanocomposite platform through DPV, which is much lower than that of the value achieved through individual SWCNT and rGO platforms. Some other carbon nanotube-graphene nanocomposite based electrochemical sensing platforms for the detection of acetaminophen [63,64,65] and dopamine [65,66] are summarized in Table 1.

### 3.4. Electrochemical Sensing of Glucose at Buckypaper

**Figure 7:**
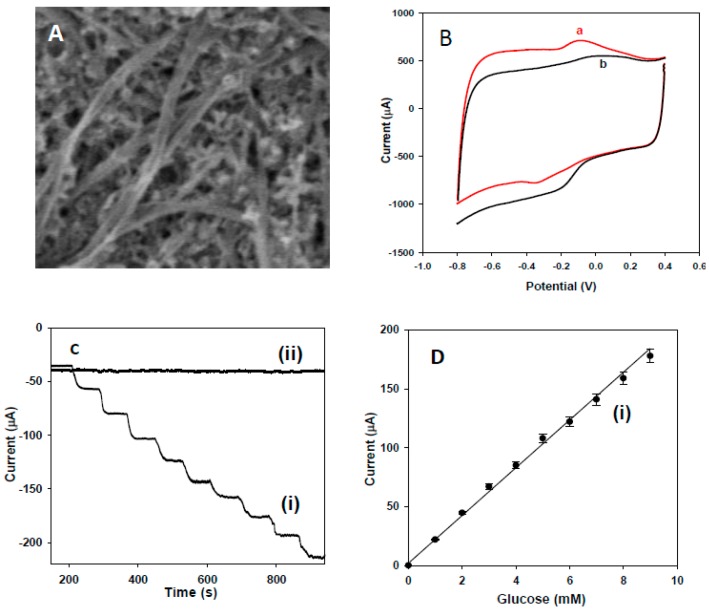
(**A**) FE-SEM images of the carbon-buckypaper. (**B**) Ti/Au/BP/GOx-HRP electrode prior to (a) and following (b) injection of 10 mM glucose at a scan rate of 10 mV/s in PBS pH 7.4. Amperometric response and calibration curve of the Ti/Au/BP/GOx-HRP (i) and Ti/Au/BP (ii) electrodes upon the addition of 1 mM glucose (**C** and **D**). All figures are reproduced with permission from [28]. Copyright 2011 Elsevier.

The morphology and composition of the buckypaper were characterized using FE-SEM. Figure 7A shows a typical SEM image of the buckypaper film, which reveals that the SWNTs were arranged in bundles that formed networks. The biosensor composed of Au/BP/GOx-HRP on a titanium surface was studied for its glucose response. Figure 7B shows the CV responses of Au/BP/GOx-HRP/Ti prior to (a) and following (b) the addition of 10 mM glucose in 0.1 M PBS (pH 7.4) at the scan rate of 10 mV/s. The CV curves revealed a decreased oxidation peak, as well as a significantly increased reduction peak, showing a strong response to glucose oxidation. The increase of the reduction peak might have been due to the H_2_O_2_ that was generated through the oxidation of glucose via the immobilized GOx [67,68,69]. The amperometric response of the Au/BP/GOx-HRP/Ti biosensor at an optimized potential under physiological pH 7.4 in 0.1 M PBS upon the successive addition of 1 mM glucose is shown in Figure 7C. For comparison, the amperometric response of the Ti/Au/BP electrode is also included in Figure 7C (Curve ii). No obvious current response was observed at the non-enzymatic electrode, while a strong response was achieved through the Au/BP/GOx-HRP/Ti biosensor (Figure 7C(i)). Figure 7D illustrates the corresponding current *vs.* different glucose concentration with an excellent linear correlation coefficient of R^2^ = 0.993. The limit of detection for the detection of glucose through this fabricated biosensor was estimated to be 0.01 mM. The detection limit obtained through this study was much lower than that of previously reported for the buckypaper based detection of glucose [70].

### 3.5. Electrocatalytic Performance of SWCNTs, rGO and SWCNTs-rGO for Acetaminophen and Valacyclovir: Comparative Analysis

To demonstrate the electrocatalytic behavior of three carbon nanomaterial-based platform nanocomposites (SWCNT, rGO, and SWCNT-rGO), the responses to acetaminophen and valacyclovir were compared. As seen in Figure 1C, Figure 3B, and Figure 6B, the electrocatalytic behavior of 50 µM acetaminophen at the SWCNT, rGO and SWCNT-rGO nanocomposite electrodes revealed significant changes in oxidation-reduction peak current responses. In the present study, the lowest oxidation peak current was observed with the rGO platform, whereas a six fold increase in the oxidation current response was observed at the SWCNT platform under identical experimental conditions. The electrocatalytic oxidation current response was greatly improved at the SWCNT-rGO nanocomposite, which exhibited an over six times higher current response than that of SWCNT platform, and was12 times higher than that of the rGO. The peak to peak separation (ΔE_p_) was estimated to be 75 mV, 45 mV, and 30 mV at the rGO, SWCNT and SWCT-rGO nanocomposites, respectively. Decreasing ΔE_p_ values, in the order rGO ˃ SWCT ˃ SWCT-rGO observed through these platforms reflected a more rapid electron transfer reaction with efficient electrocatalytic oxidation. A similar pattern was observed for the oxidation of 50 µM valacyclovir through the SWCNT (Figure 1D) and SWCT-rGO nanohybrid (Figure 6C), where we observed an almost 21% increment of oxidation current response with the SWCT-rGO nanocomposite. In general, the structure and properties of carbon materials, such as surface functional groups, graphitization structure, charge density and surface area, have a significant impact on their electrocatalytic activities. The high crystallinity and one-dimensional (1D) structure of CNTs endow high conductivity and high surface areas. Graphene, in addition to possessing similar stable physical properties as CNTs, has larger edge planes with high charge densities. Further, the heterogeneity of carbon materials with edge planes may better stabilize and augment electrocatalytic activity [71]. Due to strong van der Waals forces, 1D CNTs and two-dimensional (2D) rGO sheets are both inclined to aggregate, or stack with each other in forming a three-dimensional (3D) CNT-rGO nanocomposite network with synergetic properties [72]. As a result, this hybrid material provides exceptional characteristics with a higher nominal edge density per unit area than individual CNTs and rGO nanosheets, thereby fully utilizing it capacities for charge density, surface area, and available active sites for catalytic reactions. In fact, the integration of CNT and rGO to comprise nanocomposite materials is a quite intriguing strategy for enhancing the dispersion of CNT and rGO, toward exploiting the distinct advantages of both CNT and rGO in obtaining a robust and efficacious electronic and thermally conductive 3D network [73].

## 4. Conclusions

Carbon-based nanomaterials and composites thereof were employed in the design of highly sensitive electrochemical sensors for the detection of biologically and pharmaceutically important compounds. Carbon and its derivatives possess excellent electrocatalytic properties for the modified sensors, such as enhanced detection sensitivity, electrocatalytic effects, high conductivity, and reduced fouling. These superior attributes endow SWCNTs, graphene, and buckypaper nanocomposites with great advantages for enhanced sensing and biosensing applications. In the present feature article, important biomolecules such as methylglyoxal, glucose, and NADH, as well as pharmaceutical molecules such as acetaminophen and valacyclovir were selected to establish and verify the sensing performance of carbon nanomaterials based electrochemical platforms. Carbon based nanomaterials provide an excellent electrocatalytic platform for the sensitive detection of these compounds. Further demonstrations with acetaminophen and valacyclovir revealed that SWCNT-rGO nanocomposites possess excellent electrocatalytic activity in comparison to individual SWCNTs and rGO platforms. Among the carbon based nanomaterials tested in this study, SWCNT-rGO nanocomposites were shown to be an excellent platform for use in the core architectures of future sensor/biosensor designs. Carbon nanomaterials and nanohybrids thereof may play a critical role in the future development of advanced point of care diagnostics.

## References

[1] Kirsch J., Siltanen C., Zhou Q., Revzin A., Simonian A. (2013). Biosensor technology: Recent advances in threat agent detection and medicine. Chem. Soc. Rev..

[2] Xu R.X., Yu X.Y., Gao C., Liu J.H., Compton R.G., Huang X.J. (2013). Enhancing selectivity in stripping voltammetry by different adsorption behaviors: The use of nanostructured Mg-Al-layered double hydroxides to detect Cd (II). Analyst.

[3] Govindhan M., Lafleur T., Adhikari B.-R., Chen A. (2015). Electrochemical sensor based on carbon nanotubes for the simultaneous detection of phenolic pollutants. Electroanalysis.

[4] Hung V.W.-S., Kerman K. (2011). Gold electrodeposition on carbon nanotubes for the enhanced electrochemical detection of homocysteine. Electrochem. Commun..

[5] Govindhan M., Adhikari B.-R., Chen A. (2014). Nanomaterials-based electrochemical detection of chemical contaminants. RSC Adv..

[6] Pandey P., Datta M., Malhotra B.D. (2008). Prospects of nanomaterials in biosensors. Anal. Lett..

[7] Chen A., Chatterjee S. (2013). Nanomaterials based electrochemical sensors for biomedical applications. Chem. Soc. Rev..

[8] Figueiredo-Filho L.C.S., Brownson D.A.C., Fatibello-Filho O., Banks C.E. (2013). Exploring the origins of the apparent “electrocatalytic” oxidation of kojic acid at graphene modified electrodes. Analyst.

[9] Ahmadalinezhad A., Wu G., Keeler W., Chen A. (2014). Fabrication and electrochemical study of carbon modified TiO_2_ nanowires. Electrochem. Commun..

[10] Goyal R.N., Chatterjee S., Rana A.R.S. (2010). The effect of modifying an edge-plane pyrolytic graphite electrode with single-wall carbon nanotubes on its use for sensing diclofenac. Carbon.

[11] Revin S.B., John S.A. (2012). Electrochemical sensor for neurotransmitters at physiological pH using a heterocyclic conducting polymer modified electrode. Analyst.

[12] Ahammad A., Lee J., Rahman M. (2009). Electrochemical Sensors Based on Carbon Nanotubes. Sensors.

[13] Chatterjee S., Chen A. (2012). Functionalization of carbon buckypaper for the sensitive determination of hydrogen peroxide in human urine. Biosens. Bioelectron..

[14] Chatterjee S., Chen A. (2012). Voltammetric detection of the dicarbonyl compound: Methylglyoxal as a flavoring agent in wine and beer. Anal. Chim. Acta.

[15] Wanekaya A.K. (2011). Applications of nanoscale carbon-based materials in heavy metal sensing and detection. Analyst.

[16] Hong G., Diao S., Antaris A.L., Dai H. (2015). Carbon nanomaterials for biological imaging and nanomedicinal therapy. Chem. Rev..

[17] Ogawa S., Nakayama K., Nakayama M., Mori T., Matsushima M., Okamura M., Senda M., Nako K., Miyata T., Ito S. (2010). Methylglyoxal is a predictor in type 2 diabetic patients of intima-media thickening and elevation of blood pressure. Hypertension.

[18] Chatterjee S., Wen J., Chen A. (2013). Electrochemical determination of methylglyoxal as a biomarker in humanplasma. Biosens. Bioelectron..

[19] Shah B., Lafleur T., Chen A. (2013). Carbon nanotube based electrochemical sensor for the sensitive detection of valacyclovir. Faraday Discuss..

[20] Liu M., Chen Q., Lai C., Zhang Y., Deng J., Li H., Yao S. (2013). A double signal amplification platform for ultrasensitive and simultaneous detection of ascorbic acid, dopamine, uric acid and acetaminophen based on a nanocomposite of ferrocene thiolate stabilized Fe_3_O_4_@Au nanoparticles with graphene sheet. Biosens. Bioelectron..

[21] Vedala H., Sorescu D.C., Kotchey G.P., Star A. (2011). Chemical sensitivity of graphene edges decorated with metal nanoparticles. Nano Lett..

[22] Liu F., Xiang G., Yuan R., Chen X., Luo F., Jiang D., Huang S., Li Y., Pu X. (2014). Procalcitonin sensitive detection based on grapheneGÇôgold nanocomposite film sensor platform and single-walled carbon nanohorns/hollow Pt chains complex as signal tags. Biosens. Bioelectron..

[23] Ali I., Khan T., Omanovic S. (2014). Direct electrochemical regeneration of the cofactor NADH on bare Ti, Ni, Co and Cd electrodes: The influence of electrode potential and electrode material. J. Mol. Catal. A Chem..

[24] James L.P., Mayeux P.R., Hinson J.A. (2003). Acetaminophen-induced hepatotoxicity. Drug Metabol. Dispos..

[25] Govindhan M., Amiri M., Chen A. (2015). Au nanoparticle/graphene nanocomposite as a platform for the sensitive detection of NADH in human urine. Biosens. Bioelectron..

[26] Adhikari B.R., Govindhan M., Chen A. (2015). Sensitive detection of acetaminophen with graphene-based electrochemical sensor. Electrochim. Acta.

[27] Endo M., Muramatsu H., Hayashi T., Kim Y.A., Terrones M., Dresselhaus M.S. (2005). Nanotechnology: Buckypaper from coaxial nanotubes. Nature.

[28] Ahmadalinezhad A., Wu G., Chen A. (2011). Mediator-free electrochemical biosensor based on buckypaper with enhanced stability and sensitivity for glucose detection. Biosens. Bioelectron..

[29] Wang J., Musameh M., Lin Y. (2003). Solubilization of carbon nanotubes by nafion toward the preparation of amperometric biosensors. J. Am. Chem. Soc..

[30] Wang J., Musameh M. (2003). Enzyme-dispersed carbon-nanotube electrodes: A needle microsensor for monitoring glucose. Analyst.

[31] Hrapovic S., Liu Y., Male K.B., Luong J.H. (2004). Electrochemical biosensing platforms using platinum nanoparticles and carbon nanotubes. Anal. Chem..

[32] Rubianes M.A.D., Rivas G.A. (2005). Enzymatic biosensors based on carbon nanotubes paste electrodes. Electroanalysis.

[33] Wang H.S., Li T.H., Jia W.L., Xu H.Y. (2006). Highly selective and sensitive determination of dopamine using a nafion/carbon nanotubes coated poly(3-methylthiophene) modified electrode. Biosens. Bioelectron..

[34] Zhang Y., Cai Y., Su S. (2006). Determination of dopamine in the presence of ascorbic acid by poly (styrene sulfonic acid) sodium salt/single-wall carbon nanotube film modified glassy carbon electrode. Anal. Biochem..

[35] Habibi B., Jahanbakhshi M., Pournaghi-Azar M.H. (2011). Simultaneous determination of acetaminophen and dopamine using SWCNT modified carbonGÇôceramic electrode by differential pulse voltammetry. Electrochim. Acta.

[36] Zeng B., Wei S., Xiao F., Zhao F. (2006). Voltammetric behavior and determination of rutin at a single-walled carbon nanotubes modified gold electrode. Sens. Actuators B Chem..

[37] Yu X., Kim S.N., Papadimitrakopoulos F., Rusling J.F. (2005). Protein immunosensor using single-wall carbon nanotube forests with electrochemical detection of enzyme labels. Mol. Biosyst..

[38] Li J., Zhang Y., Yang T., Zhang H., Yang Y., Xiao P. (2009). DNA biosensor by self-assembly of carbon nanotubes and DNA to detect riboflavin. Mater. Sci. Eng. C.

[39] Yogeswaran U., Thiagarajan S., Chen S.M. (2007). Nanocomposite of functionalized multiwall carbon nanotubes with nafion, nano platinum, and nano gold biosensing film for simultaneous determination of ascorbic acid, epinephrine, and uric acid. Anal. Biochem..

[40] Yi H., Zheng D., Hu C., Hu S. (2008). Functionalized multiwalled carbon nanotubes through *in situ* electropolymerization of brilliant cresyl blue for determination of epinephrine. Electroanalysis.

[41] Guo M., Chen J., Li J., Nie L., Yao S. (2004). Carbon nanotubes-based amperometric cholesterol biosensor fabricated through layer-by-layer technique. Electroanalysis.

[42] Li G., Liao J.M., Hu G.Q., Ma N.Z., Wu P.J. (2005). Study of carbon nanotube modified biosensor for monitoring total cholesterol in blood. Biosens. Bioelectron..

[43] Martinez N.A., Messina G.A., Bertolino F.A., Salinas E., Raba J. (2008). Screen-printed enzymatic biosensor modified with carbon nanotube for the methimazole determination in pharmaceuticals formulations. Sens. Actuators B Chem..

[44] Ghalkhani M., Shahrokhian S., Ghorbani-Bidkorbeh F. (2009). Voltammetric studies of sumatriptan on the surface of pyrolytic graphite electrode modified with multi-walled carbon nanotubes decorated with silver nanoparticles. Talanta.

[45] Kachoosangi R.T., Wildgoose G.G., Compton R.G. (2008). Sensitive adsorptive stripping voltammetric determination of paracetamol at multiwalled carbon nanotube modified basal plane pyrolytic graphite electrode. Anal. Chim. Acta.

[46] Hsu Y.W., Hsu T.K., Sun C.L., Nien Y.T., Pu N.W., Ger M.D. (2012). Synthesis of CuO/graphene nanocomposites for nonenzymatic electrochemical glucose biosensor applications. Electrochim. Acta.

[47] Luo J., Jiang S., Zhang H., Jiang J., Liu X. (2012). A novel non-enzymatic glucose sensor based on Cu nanoparticle modified graphene sheets electrode. Anal. Chim. Acta.

[48] Alwarappan S., Liu C., Kumar A., Li C.Z. (2010). Enzyme-doped graphene nanosheets for enhanced glucose biosensing. J. Phys. Chem. C.

[49] Sun C.L., Lee H.H., Yang J.M., Wu C.C. (2011). The simultaneous electrochemical detection of ascorbic acid, dopamine, and uric acid using graphene/size-selected Pt nanocomposites. Biosens. Bioelectron..

[50] Raj M.A., John S.A. (2014). Graphene layer modified glassy carbon electrode for the determination of norepinephrine and theophylline in pharmaceutical formulations. Anal. Methods.

[51] Tabrizi M.A., Azar S.A., Varkani J.N. (2014). Eco-synthesis of graphene and its use in dihydronicotinamide adenine dinucleotide sensing. Anal. Biochem..

[52] Li L., Lu H., Deng L. (2013). A sensitive NADH and ethanol biosensor based on graphene-Au nanorods nanocomposites. Talanta.

[53] Fan Y., Yang X., Yang C., Liu J. (2012). Au-TiO_2_/Graphene nanocomposite film for electrochemical sensing of hydrogen peroxide and NADH. Electroanalysis.

[54] Wang K., Wu J., Liu Q., Jin Y., Yan J., Cai J. (2012). Ultrasensitive photoelectrochemical sensing of nicotinamide adenine dinucleotide based on graphene-TiO_2_ nanohybrids under visible irradiation. Anal. Chim. Acta.

[55] Sheng Z.H., Zheng X.Q., Xu J.Y., Bao W.J., Wang F.B., Xia X.H. (2012). Electrochemical sensor based on nitrogen doped graphene: Simultaneous determination of ascorbic acid, dopamine and uric acid. Biosens. Bioelectron..

[56] Du J., Yue R., Yao Z., Jiang F., Du Y., Yang P., Wang C. (2013). Nonenzymatic uric acid electrochemical sensor based on graphene-modified carbon fiber electrode. Colloids Surf. A.

[57] Kaur B., Pandiyan T., Satpati B., Srivastava R. (2013). Simultaneous and sensitive determination of ascorbic acid, dopamine, uric acid, and tryptophan with silver nanoparticles-decorated reduced graphene oxide modified electrode. Colloids Surf. B.

[58] Xu T.Q., Zhang Q.L., Zheng J.N., Lv Z.Y., Wei J., Wang A.J., Feng J.J. (2014). Simultaneous determination of dopamine and uric acid in the presence of ascorbic acid using Pt nanoparticles supported on reduced graphene oxide. Electrochim. Acta.

[59] Raj M.A., John S.A. (2013). Simultaneous determination of uric acid, xanthine, hypoxanthine and caffeine in human blood serum and urine samples using electrochemically reduced graphene oxide modified electrode. Anal. Chim. Acta.

[60] Liu G.T., Chen H.F., Lin G.M., Ye P., Wang X.P., Jiao Y.Z., Guo X.Y., Wen Y., Yang H.F. (2014). One-step electrodeposition of graphene loaded nickel oxides nanoparticles for acetaminophen detection. Biosens. Bioelectron..

[61] Zheng M., Gao F., Wang Q., Cai X., Jiang S., Huang L., Gao F. (2013). Electrocatalytical oxidation and sensitive determination of acetaminophen on glassy carbon electrode modified with graphene-chitosan composite. Mater. Sci. Eng. C.

[62] Kang X., Wang J., Wu H., Liu J., Aksay I.A., Lin Y. (2010). A graphene-based electrochemical sensor for sensitive detection of paracetamol. Talanta.

[63] Chen X., Zhu J., Xi Q., Yang W. (2012). A high performance electrochemical sensor for acetaminophen based on single-walled carbon nanotube-graphene nanosheet hybrid films. Sens. Actuators B Chem..

[64] Arvand M., Gholizadeh T.M. (2013). Simultaneous voltammetric determination of tyrosine and paracetamol using a carbon nanotube-graphene nanosheet nanocomposite modified electrode in human blood serum and pharmaceuticals. Colloids Surf. B.

[65] Cheemalapati S., Palanisamy S., Mani V., Chen S.M. (2013). Simultaneous electrochemical determination of dopamine and paracetamol on multiwalled carbon nanotubes/graphene oxide nanocomposite-modified glassy carbon electrode. Talanta.

[66] Sun C.L., Chang C.T., Lee H.H., Zhou J., Wang J., Sham T.K., Pong W.F. (2011). Microwave-assisted synthesis of a core shell MWCNT/GONR heterostructure for the electrochemical detection of ascorbic acid, dopamine, and uric acid. ACS Nano.

[67] Liu Y., Wang M., Zhao F., Xu Z., Dong S. (2005). The direct electron transfer of glucose oxidase and glucose biosensor based on carbon nanotubes/chitosan matrix. Biosens. Bioelectron..

[68] Reilly C.A., Aust S.D. (1997). Peroxidase substrates stimulate the oxidation of hydralazine to metabolites which cause single-strand breaks in DNA. Chem. Res. Toxicol..

[69] Rodriguez-Lopez J.N., Lowe D.J., Hernaíndez-Ruiz J., Hiner A.N.P., Garcia-Caínovas F., Thorneley R.N.F. (2001). Mechanism of reaction of hydrogen peroxide with horseradish peroxidase: Identification of intermediates in the catalytic cycle. J. Am. Chem. Soc..

[70] Papa H., Gaillard M., Gonzalez L., Chatterjee J. (2014). Fabrication of functionalized carbon nanotube buckypaper electrodes for application in glucose biosensors. Biosensors.

[71] Tourani S., Rashidi A., Safekordi A., Aghabozorg H.R., Khorasheh F. (2015). Synthesis of reduced graphene oxide—Carbon nanotubes (rGO-CNT) composite and its use as a novel catalyst support for hydro-purification of crude terephtalic acid. Ind. Eng. Chem. Res..

[72] Zhang C., Ren L., Wang X., Liu T. (2010). Graphene oxide-assisted dispersion of pristine multiwalled carbon nanotubes in aqueous media. J. Phys. Chem. C.

[73] Stoner R.B., Glass T.J. (2012). Carbon nanostructes: A morphological classification for charge density optimization. Diam. Relat. Mater..

